# Adenosine-to-Inosine RNA Editing Within Corticolimbic Brain Regions Is Regulated in Response to Chronic Social Defeat Stress in Mice

**DOI:** 10.3389/fpsyt.2019.00277

**Published:** 2019-04-26

**Authors:** Alec L. W. Dick, Khen Khermesh, Evan Paul, Fabian Stamp, Erez Y. Levanon, Alon Chen

**Affiliations:** ^1^Department of Stress Neurobiology and Neurogenetics, Max Planck Institute of Psychiatry, Munich, Germany; ^2^CytoReason, Tel-Aviv, Israel; ^3^The Mina and Everard Goodman Faculty of LifeSciences, Bar-Ilan University, Ramat-Gan, Israel; ^4^Department of Neurobiology, Weizmann Institute of Science, Rehovot, Israel

**Keywords:** A-to-I RNA editing, chronic social defeat stress, microfluidics-based multiplex polymerase chain reaction, adenosine deaminases acting on RNA, ADAR, chronic stress

## Abstract

Adenosine-to-inosine (A-to-I) RNA editing is a co-/posttranscriptional modification of double-stranded RNA, catalyzed by the adenosine deaminase acting on RNA (ADAR) family of enzymes, which results in recognition of inosine as guanosine by translational and splicing machinery causing potential recoding events in amino acid sequences. A-to-I editing is prominent within brain-specific transcripts, and dysregulation of editing at several well-studied loci (e.g., *Gria2*, *Htr2c*) has been implicated in acute and chronic stress in rodents as well as neurological (e.g., Alzheimer’s) and psychopathological disorders such as schizophrenia and major depressive disorder. However, only a small fraction of recoding sites has been investigated within the brain following stress, and our understanding of the role of RNA editing in transcriptome regulation following environmental stimuli remains poorly understood. Thus, we aimed to investigate A-to-I editing at hundreds of loci following chronic social defeat stress (CSDS) in mice within corticolimbic regions responsive to chronic stress regulation. Adult male mice were subjected to CSDS or control conditions for 21 days and dynamic regulation of A-to-I editing was investigated 2 and 8 days following the final defeat within both the medial prefrontal cortex (mPFC) and basolateral amygdala (BLA). Employing a targeted resequencing approach, which utilizes microfluidics-based multiplex polymerase chain reaction (PCR) coupled with next-generation sequencing, we analyzed A-to-I editing at ∼100 high-confidence editing sites within the mouse brain. CSDS resulted in acute regulation of transcripts encoding several ADAR enzymes, which normalized 8 days following the final defeat and was specific for susceptible mice. In contrast, sequencing analysis revealed modest and dynamic regulation of A-to-I editing within numerous transcripts in both the mPFC and BLA of resilient and susceptible mice at both 2 and 8 days following CSDS with minimal overlap between regions and time points. Editing within the *Htr2c* transcript and relative abundance of *Htr2c* messenger RNA (mRNA)variants were also observed within the BLA of susceptible mice 2 days following CSDS. These results indicate dynamic RNA editing within discrete brain regions following CSDS in mice, further implicating A-to-I editing as a stress-sensitive molecular mechanism within the brain of potential relevance to resiliency and susceptibility to CSDS.

## Introduction

Adenosine-to-inosine (A-to-I) RNA editing is a co-/posttranscriptional modification of double-stranded RNA (dsRNA), which is catalyzed by the adenosine deaminases acting on RNA (ADAR) family of enzymes and is the most abundant form of RNA editing in higher eukaryotes (1). ADAR enzymes deaminate adenosine bases to inosine, which is recognized as guanosine by ribosomes and splicing machinery. As such, RNA editing can induce nonsynonymous amino acid changes resulting in differential protein isoform expression and thus is considered a key mechanism of transcriptome and proteome diversification in metazoans (2).

Three members of the ADAR family are encoded in the mammalian genome including the catalytically active ADAR (ADAR1) and ADARB1 (ADAR2), as well as ADARB2 (ADAR3), which lacks a catalytic domain and is primarily restricted to low-level expression within the brain (3). Furthermore, ADAR has two distinct splicing isoforms including the constitutive 110-kDa isoform ADAR1 (p110) and the interferon-inducible isoform ADAR (p150) (4). ADARs mediate RNA editing at millions of sites in the mammalian transcriptome in both coding and noncoding RNA. Recent evidence suggests that ADAR primarily mediates A-to-I editing at repetitive elements, such as *Alu* repeats in primates, where ADARB1 is primarily responsible for editing at coding sites, although a degree of overlap exists between targeted sites of the two enzymes within mammalian tissues (5). Although several functions are known for editing events in noncoding sites [e.g., alteration of microRNA (miRNA) binding to 3′untranslated regions (UTRs) and alternative splicing regulation] (6–8), much interest has been focused on editing sites within coding regions capable of inducing nonsynonymous recoding events. These events are appreciated as a common form of proteome diversification in both basal and pathological states (9). Interestingly, such recoding events are enriched within the brain and also reside more commonly in transcripts associated with brain function, such as those encoding ion channels and neuromodulator receptors (10). For example, well-established ADARB1-dependent editing of the *Gria2* transcript encoding the alpha-amino-3-hydroxy-5-methyl-4-isoxazole propionate (AMPA) receptor subunit Glutamate Ionotropic Receptor AMPA Type Subunit 2 (GRIA2) is essential for normal development as *Adarb1* knockout mice die within 3 weeks of birth. This can be rescued upon transgenic coexpression of the fully edited *Gria2* isoform in these mice (11). Another well-established role of RNA editing within the mammalian brain is regulation of the 5-hydroxytryptamine_2C_ (5-HT_2C_) receptor as multiple recoding sites in the *Htr2c* transcript generates multiple 5-HT_2C_ receptor isoforms with varying G protein affinities and thus receptor function (12).

RNA editing within the brain is also sensitive to environmental and pharmacological stimuli as acute and chronic stress as well as antidepressant treatment in rodents dynamically regulates *Htr2c* editing and thus serotonergic signaling within discrete brain regions (13, 14). Moreover, editing of the *Htr2c* transcript is observed within both the dorsolateral prefrontal cortex (PFC) (BA9) and anterior cingulate cortex (BA24) of patients with major depressive disorder (MDD), indicative of the translational relevance of investigating stress-induced regulation of well-conserved editing sites in rodent models of acute and chronic stress (15, 16). Despite this, our understanding of the stress-induced changes in the RNA editome remain restricted to well-studied candidate loci. Broader high-throughput approaches are necessary to identify novel stress-sensitive editing sites within the brain of potential relevance to stress-related psychiatric disorders.

Recent advancements in next-generation sequencing (NGS) technologies have significantly enhanced our ability to accurately quantify A-to-I editing throughout the transcriptome and broadened our understanding of aberrant A-to-I editing in several neurological diseases (e.g., Alzheimer’s disease, epilepsy, and amyotrophic lateral sclerosis) (17–19) and psychiatric disorders (schizophrenia, bipolar disorder, and autism spectrum disorder) (20). However, application of NGS-based techniques in rodent models of acute and chronic stress is lacking such that our understanding of the role of RNA editing in acute and long-term adaptations to stress remains poorly understood.

Thus, this study aimed to investigate aberrant RNA editing within corticolimbic brain regions following chronic social defeat stress (CSDS) in adult mice. CSDS is a well-characterized model of depression-like behavior with significant etiological, predictive, discriminative, and face validity. CSDS induces diverse transcriptome changes within corticolimbic circuits such as the medial prefrontal cortex (mPFC) and basolateral amygdala (BLA) thought to subserve stable behavioral deficits in this model (21, 22). We therefore hypothesized that CSDS would induce dynamic changes within the RNA editome within the mPFC and BLA. To identify novel stress-sensitive editing sites within these brain regions, we employed an established microfluidics-based multiplex polymerase chain reaction and deep sequencing (mmPCR-seq) approach (19, 23) to accurately quantify RNA editing at hundreds of loci within the brain following CSDS.

## Methods

### Animals

Male C57BL/6 mice at 10 to 12 weeks old were employed for all experiments (Charles River, Sulzfeld, Germany). Mice were single housed in the animal facilities of the Max Planck Institute of Psychiatry in Munich, Germany, for 1 week prior to experimentation and were maintained under standard conditions (12L:12D light cycle, lights on at 07:00 AM, temperature 23 ± 2°C) with food and water available *ad libitum*. All experiments were approved by and conducted in accordance with the regulations of the local Animal Care and Use Committee (Government of Upper Bavaria, Munich, Germany).

### Chronic Social Defeat Stress

Mice were randomly assigned to stress (*n* = 25) or control conditions (*n* = 16) and were subjected to 21 days of social defeat or daily handling, respectively. CSDS was conducted essentially as previously described (24). Briefly, male CD1 mice (16 to 18 weeks of age) were employed as resident mice and were habituated to the social defeat cage for 1 week prior to experimentation. On each defeat day, experimental mice were introduced into the home cage (45 cm × 25 cm) of a dominant resident mouse, and mice were left until a social defeat was achieved with minimal injury to experimental mice (generally >2 min). Once mice were defeated, the animals were separated by a wire mesh to prevent physical contact while enabling sensory contact for 24 h. Experimental mice were defeated daily by an unfamiliar, resident mouse for 21 days between 9:00 and 16:00 h to minimize habituation to the CSDS procedure. Control mice (Con) were singly housed in their home cage and handled daily throughout the CSDS procedure. Immediately following the final defeat, experimental mice were single housed. One day following the final defeat, all mice were subjected to the social interaction (SI) test to enable classification of susceptible (SUS) [social interaction (SI) ratio <1] and resilient mice (RES) (SI ratio >1) as previously described (22, 24). Following the SI test, all mice remained in their home cage undisturbed until tissue collection either 2 days (control, *n* = 8; susceptible, *n* = 10; resilient, *n* = 4) or 8 days (control, *n* = 8; susceptible, *n* = 8; resilient, *n* = 3) following the final defeat.

### Tissue Collection and Corticosterone Analysis

Either 2 or 8 days following the final defeat, mice were sacrificed (9:00–10:00) and brains were rapidly dissected, snap frozen, and stored at −80°C. Bilateral adrenal glands were dissected, cleaned of excess fat, and weighed. Whole blood was collected in Ethylenediaminetetraacetic acid tubes and centrifuged at 8,000 × g for 10 min at 4°C. Plasma was then aliquoted and stored at −20°C until corticosterone analysis, employing a commercially available radioimmunoassay kit (MP Biomedicals Inc).

### Brain Region Microdissection, RNA Extraction, and Reverse Transcription

Frozen brains were serially sectioned at 250 µm on a cryostat, and the mPFC (including prelimbic, infralimbic, and cingulate cortex) and BLA were microdissected and stored at −80°C. Total RNA was extracted from mPFC and BLA tissue from each animal utilizing the miRNeasy mini kit (QIAGEN). Briefly, tissue punches were lysed in 700 µl of Qiazol, and total RNA was extracted as per manufacturer’s instructions (QIAGEN). RNA integrity was assessed on an Agilent Tapestation 2200 with the RNA screentape kit (Thermo Fisher), and all RNA samples were confirmed to have RNA integrity number (RIN) values > 8. Total RNA was DNase treated with the TURBO DNase free kit as per manufacturer’s instructions (Ambion). DNase-treated RNA was quantified with the Qubit 3.0 (Thermo Fisher), and 200 ng of RNA was reverse transcribed to cDNA in 20-µL reactions employing the iScript™ cDNA Synthesis Kit as per manufacturer’s instructions (Bio-Rad). Complementary DNA (cDNA) was stored at −20°C prior to further analysis. Due to failure of cDNA synthesis for multiple samples, final group sizes analyzed for RNA editing and quantitative PCR (qPCR) analysis were as follows: control 2 days, *n* = 6–8; susceptible 2 days, *n* = 10; resilient 2 days, *n* = 4; control 8 days, *n* = 5–8; susceptible 8 days, *n* = 7–8; resilient 8 days, *n* = 3.

### Quantitative Real-Time Polymerase Chain Reaction

Determination of relative transcript expression was conducted using the 2^−ΔΔCT^ method (25, 26). Exon spanning primers for candidate transcripts *Adar*, *Adar variant 2*, *Adar variant 3*, *Adarb1*, *Adarb2*, *Nova1*, *Commd2*, *Gria4*, and *Htr2c* and endogenous controls *Gapdh*, *Rpl13a*, and *Sdha* were designed using Primer 3 (http://frodo.wi.mit.edu/). Primer efficiencies were confirmed to be 90–110%. qPCR was conducted on a Quantistudio Flex7 PCR system (Applied Biosystems, USA) using Quantifast SYBR^®^ Green (QIAGEN) as per manufacturer’s instructions. All data are expressed as fold change relative to control mice at each time point.

### Targeted Resequencing of RNA Editing Sites in RNA Samples Using the Fluidigm Access Array Coupled With Illumina HiSeq 2500 Sequencing

To precisely detect and measure the levels of A-to-I RNA editing at candidate editing sites in mouse brain tissue, we employed a targeted resequencing approach utilizing mmPCR-seq essentially as previously described (19). Candidate editing sites were selected from the mouse RADAR database (v.2; http://rnaedit.com/) based on the following criteria: i) location within protein coding genes, ii) induction of nonsynonymous amino acid changes, iii) species conservation, and iv) location within genes associated with neuronal function. Applying these criteria, we generated a candidate list of 551 editing sites for which targeted gene and editing-site-specific exon spanning primers were designed using Primer 3.0 (http://frodo.wi.mit.edu/). Selected primers were tested for specificity and sensitivity by PCR prior to their inclusion to the finalized primer set (**Supplemental Table 1**). Amplification of target regions containing targeting editing loci was conducted with the Access Array™ System for Illumina Sequencing Systems as per manufacturer’s instructions. Briefly, 4 µL of single primer pair (4 μM per primer in 1× AA loading buffer) was loaded into the primer inlets of the 48.48 Access Array integrated fluidic circuits (IFC) (Fluidigm). To prepare the cDNA templates, 2.25 μL of each cDNA sample was added to 2.75 μL of presample mix containing the following enzyme and reagents from the Roche FastStart High Fidelity PCR System: 0.5 μL of 10× FastStart High Fidelity Reaction Buffer wo/Mg, 0.5 μL of dimethyl sulfoxide (DMSO) (5%), 0.1 μL of 10 mM PCR Grade Nucleotide Mix (200 μM), 0.9 μL of 25 mM MgCl_2_ (4.5 mM), 0.25 μL of 20× Access Array Loading Reagent (Fluidigm), 0.05 μL of FastStart High Fidelity Enzyme Blend, and 0.7 μL of PCR grade water. Four microliters of this mix was loaded into the sample inlets of the 48.48 Access Array IFC (Fluidigm). After the loading of both samples and primers *via* IFC Controller AX (Fluidigm) loading script, the IFC was subject to thermal cycling using the FC1 Cycler (Fluidigm) with the following program for 40 cycles: 50°C for 2 min, 70°C for 20 min, 95°C 10 min; 10 cycles of 95°C for 15 s, 59.5°C for 30 s, 72°C for 1 min; 4 cycles of 95°C for 15 s, 80°C for 30 s, 59.5°C for 30 s, 72°C for 1 min; 10 cycles of 95°C for 15 s, 59.5°C for 30 s, 72°C for 1 min; 4 cycles of 95°C for 15 s, 80°C for 30 s, 60°C for 30 s, 72°C for 1 min; 8 cycles of 95°C for 15 s, 59.5°C for 30 s, 72°C for 1 min; 4 cycles of 95°C for 15 s, 80°C for 30 s, 60°C for 30 s; 72°C for 1 min; finalizing with 72°C for 3 min. Once PCR has terminated, the IFC was transferred to another IFC Controller AX (Fluidigm) and mini-libraries were harvested by the controller harvest script. Thus, mini-libraries from each sample were obtained for further barcoding and sequencing.

### Sequencing Adaptor and Barcode Addition

For each sample, 1.0 μL of the PCR products harvested from the IFC was 1:110 diluted and added to 15 μL of presample mix containing the following enzyme and reagents from the Roche FastStart High Fidelity PCR System: 2 μL of 10× FastStart High Fidelity Reaction Buffer wo/Mg, 1 μL of DMSO (5%), 0.4 μL of 10 mM PCR Grade Nucleotide Mix (200 μM), 3.6 μL of 25 mM MgCl_2_ (4.5 mM), 0.2 μL of FastStart High Fidelity Enzyme Blend, and 7.8 μL of PCR-grade water. To that samples mix, 4 μL of primer mix from the 2 μM Access Array Barcode Library for Illumina Sequencer—384 (Fluidigm, PN 100-3771), utilizing the B-set; PE2_BC_CS2 and PE1–CS1 barcode primer combination. We used the following PCR program: 95°C for 10 min; 10 cycles of 95°C for 30 s, 60°C for 30 s, and 72°C for 1 min; and 72°C for 5 min. Amplified libraries were purified with AMPure XP beads (Beckman Coulter) and subjected to paired-end 100-bp sequencing on an Illumina Hiseq2500.

### Bioinformatics Sequence Analysis

Bioinformatics analysis was conducted essentially as previously described (19). Briefly, the University of California, Santa Cruz (UCSC) mouse genome browser (NCBI37/mm9) assembly is used to identify any A/G mismatches within the target cDNA sequences. Such mismatches were summed and calculated for their signal strength according to the overall number of reads coverage and the percentage of A-to-G levels.

#### Prealignment Processing

Fastq files were deindexed into 48 samples according to the individual barcodes used by an in-house script. All reads were trimmed of the universal CS1 and CS2 sequences, and short reads (<20 nt) were removed. Alignment of the processed reads was made using bwa version 0.7.4-r385, using the mem option and the parameters -k 20 -B 3 -O 3 -T 20 for seed in the length of the average primer and for considering the Ion typical error of small indel.

##### Alignment Process

Sequences were aligned to the mouse refseq database, and reads aligning to multiple loci were excluded from further analysis. Samtools mpileup was employed for aligned sequencing reads, and in-house scripts were employed to transfer the results to the genomic locations from the refseq loci followed by counting the number of different nucleotides in each genomic location that had a *q* score ≥20. To obtain high-confidence editing sites, only loci with >2,000 reads were included for further analysis, resulting in a total of 100 sites within the BLA and 105 sites within the mPFC (**Supplemental Table 2**). Data presented for each editing loci represent the total number of reads, and the calculated percentage of reads that have a “G” at the specified genomic location was conducted accordingly with the formula (# of “G” reads/[# of “G” reads + # of “A” reads]).

### Statistical Analysis

Two-way repeated-measures analysis of variance (ANOVA) with Sidak *post hoc* comparison was used to compare body weights throughout the CSDS procedure and SI time in the SI test between control and stressed mice. One-way ANOVAs with Sidak *post hoc* comparisons were used to compare SI ratios between control, susceptible, and resilient mice as well as between groups at each time point for body weights at sacrifice, adrenal weights, corticosterone levels, qPCR expression levels, *Htr2c* editing, and isoform abundance. Editing levels were roughly normally distributed, and no normalization was applied such that paired *t* tests were employed for each RNA editing site. Significance was accepted as *p* < 0.05. Data are presented as mean ± standard error of the mean (SEM) unless otherwise stated.

## Results

**Figure 1:**
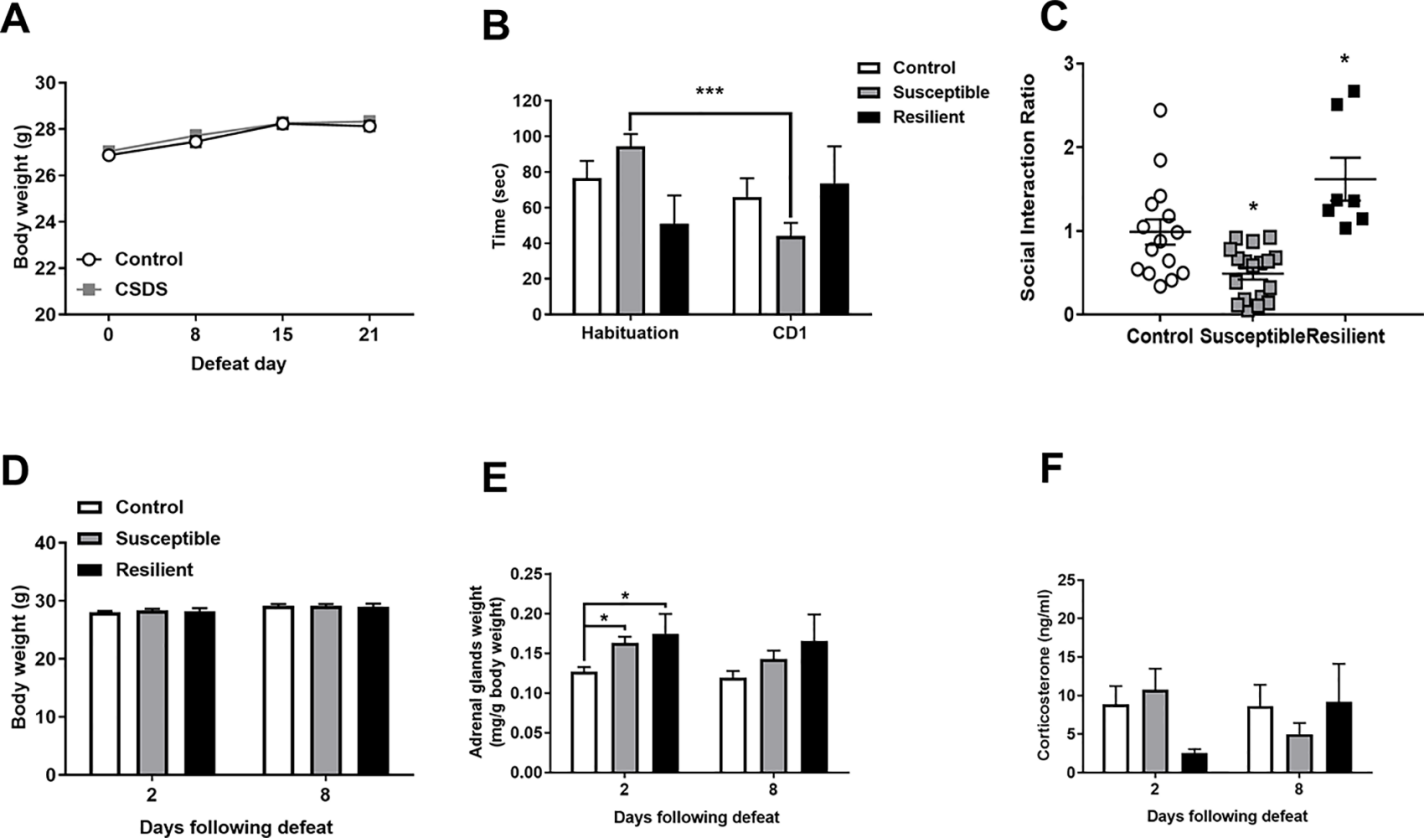
Chronic social defeat stress (CSDS) induces social avoidance and adrenal hypertrophy without changes in basal corticosterone levels. Body weight throughout the stress procedure did not differ between groups **(A)**. Following CSDS, susceptible mice spent significantly less time investigating a novel CD1 mouse than an empty cage in the social interaction (SI) test **(B)** and significantly decreased the SI ratio in susceptible and increased SI ratio in resilient mice compared to controls **(C)**. CSDS did not alter body weight at 2 or 8 days following the final defeat **(D)** but induced an increase in adrenal weight in both susceptible and resilient mice at 2 days but not 8 days **(E)** with no changes in basal corticosterone **(F)**. ***p < 0.001, two-way repeated measures (RM) ANOVA with Sidak *post hoc* comparisons; *p < 0.05, susceptible and resilient groups compared to control, one-way ANOVA with Sidak *post hoc* comparisons.

Adult male mice were subjected to 21 days of CSDS with no significant differences in body weight observed throughout the stress procedure as revealed by a main effect of time [*F*_(3,117)_ = 45.83, *p* < 0.001] but not of treatment or an interaction between factors (**Figure 1A**). CSDS significantly decreased SI with a novel CD1 mouse as evidenced by a main effect of trial [*F*_(1,37)_ = 5.252] and a treatment by trial interaction [*F*_(2,37)_ = 14.47] whereby susceptible mice spent significantly decreased time in the interaction zone in the CD1 compared to the habituation trial (*p* < 0.001, **Figure 1B**). For the SI ratio, there was a main effect of treatment [*F*_(2,37)_ = 13.72, *p* < 0.001] and significantly decreased SI ratio in susceptible mice and an increased SI ratio in resilient mice compared to controls (*p* < 0.05, **Figure 1C**). There were no differences in body weight between groups 2 or 8 days following the final defeat (**Figure 1D**). However, CSDS induced adrenal hypertrophy as evidenced by a main effect of treatment at 2 days [*F*_(2,19)_ = 5.42, *p* = 0.014] with significantly increased adrenal weights in both susceptible and resilient mice compared to controls at 2 days (*p* < 0.01) but not 8 days [*F*_(2,16)_ = 2.524, *p* = 0.111] following the final defeat (**Figure 1E**). No differences in basal corticosterone were observed at either time point following CSDS (**Figure 1F**).

**Figure 2:**
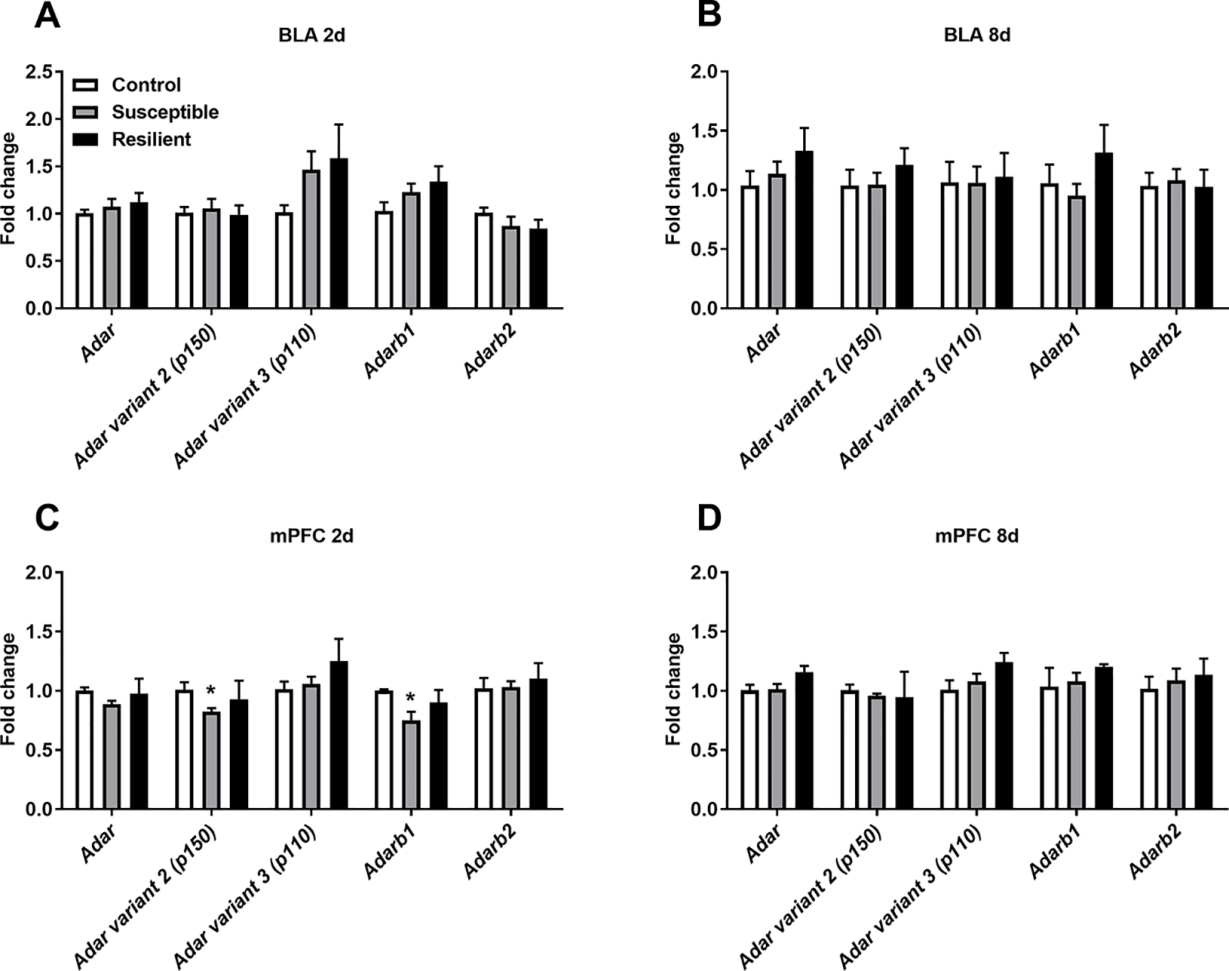
CSDS acutely alters transcripts encoding adenosine deaminases acting on RNA (ADARs) within the medial prefrontal cortex (mPFC) of susceptible mice. Following CSDS, no changes in Adar, Adarb1, or Adarb2 mRNA levels were observed within the basolateral amygdala (BLA) at 2 days **(A)** or 8 days **(B)**. CSDS significantly decreased the expression levels of Adar variant 2 (p150) and Adarb1 mRNA in the mPFC only in mice susceptible to the CSDS procedure **(C)**. No changes were observed within the mPFC 8 days following CSDS **(D)**. Control 2 days, *n* = 7; susceptible 2 days, *n* = 10; resilient 2 days, *n* = 4; control 8 days, *n* = 5–7; susceptible 8 days, *n* = 7; resilient 8 days, *n* = 3. *p < 0.05, one-way ANOVA with Sidak *post hoc* comparisons.

As stress is known to alter A-to-I editing and ADAR levels within the rodent brain, we initially analyzed mRNA expression of transcripts encoding ADAR enzymes within the BLA and mPFC following CSDS. No significant difference in Adar mRNA expression levels was observed in the BLA 2 days following the final defeat, although a trend toward increased Adar transcript variant 3 mRNA encoding the shorter ADAR1 p110 protein and Adarb1 mRNA expression was evident in both susceptible and resilient mice, yet this did not reach statistical significance (**Figure 2A**). In contrast, CSDS induced a significant decrease in Adar transcript variant 2 mRNA (*p* = 0.018) encoding the longer ADAR1 p150 protein and Adarb1 mRNA expression (*p* = 0.002) specifically in the mPFC of susceptible mice 2 days following the final defeat (**Figure 2C**). No differences in expression levels of Adar mRNAs were observed 8 days following CSDS (**Figure 2**).

To assess CSDS-induced A-to-I RNA editing within the mPFC and BLA, we employed an established mmPCR-seq approach for sensitive and accurate quantification of RNA editing (19). Employing high-confidence editing sites (see Methods), we quantified editing at 100 sites within the mouse BLA and 105 sites within the mouse mPFC. Sequencing analysis revealed that global levels of A-to-I editing within the BLA did not differ at either 2 days [Control mice (Con), 22.96% ± 0.23%; susceptible mice (SUS), 23.10% ± 0.46%; resilient mice (RES), 23.06% ± 0.33%] or 8 days (Con, 23.42% ± 0.29%; SUS, 23.09% ± 0.08%; RES, 23.39% ± 0.19%) following CSDS. Similarly, no differences were observed in the mPFC at 2 days (Con, 24.65% ± 0.24%; SUS, 24.66% ± 0.34%, RES, 25.03% ± 0.40%) or 8 days following CSDS (Con, 24.93% ± 0.25%; SUS, 24.65% ± 0.20%; RES, 24.62% ± 0.34%). Considering the lack of differences in global editing levels between groups, we pooled all samples in each region and found a significantly increased global editing level within the mPFC (24.74% ± 0.12%) compared to BLA (23.20% ± 0.14%, *p* < 0.001). This finding and global editing levels are in line with those previously reported for the rodent brain with mmPCR-seq (5, 27).

**Figure 3:**
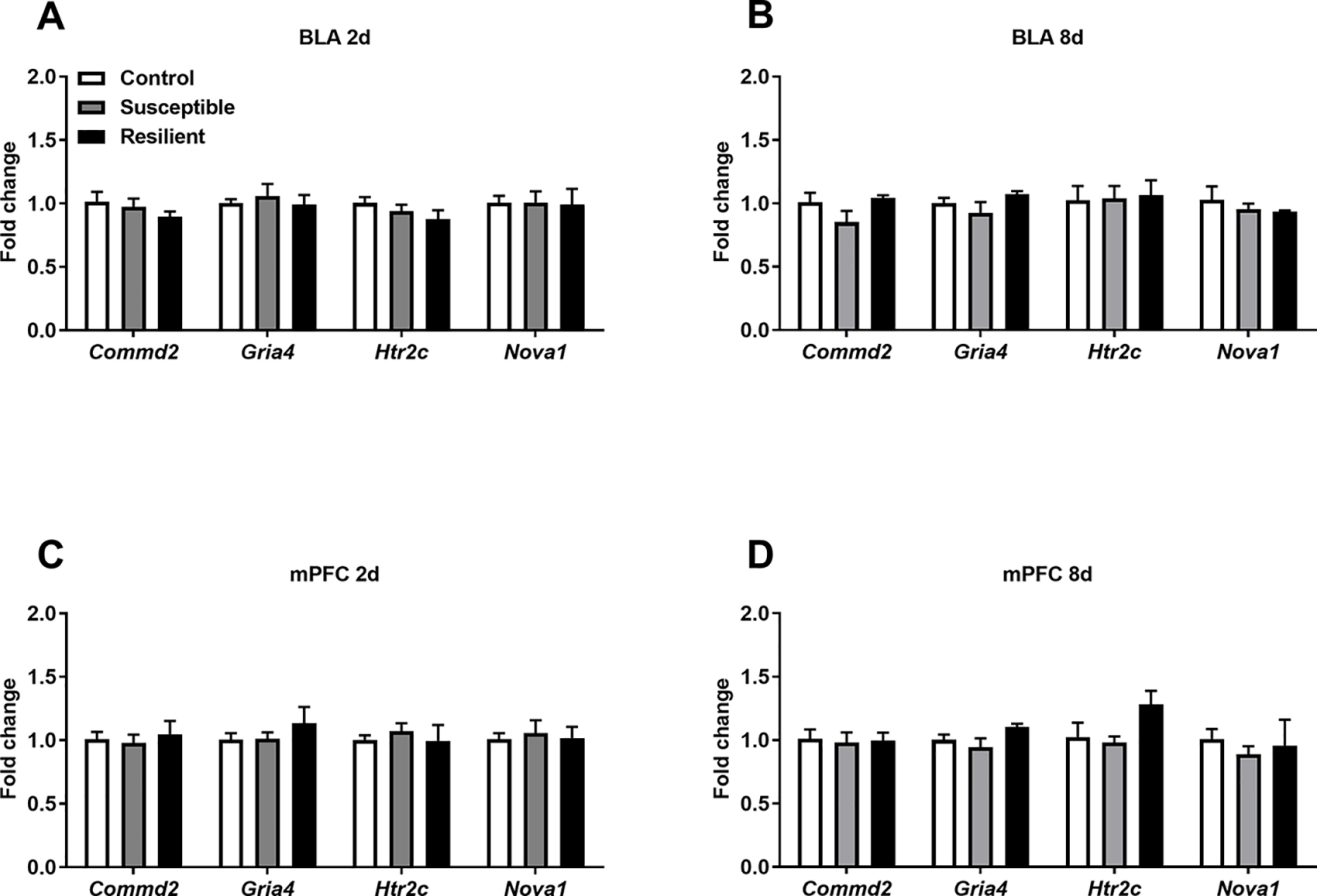
Expression levels of differentially edited transcripts are unaltered following CSDS. No significant changes in the expression levels of several differentially edited transcripts were observed with the BLA **(A)** or mPFC **(B)** at 2 days or within the BLA **(C)** or mPFC **(D)** 8 days following the final defeat. Control 2 days, *n* = 7; susceptible 2 days, *n* = 10; resilient 2 days, *n* = 4; control 8 days, *n* = 5–7; susceptible 8 days, *n* = 7; resilient 8 days, *n* = 3.

In contrast, differential A-to-I editing was observed at four editing sites in susceptible mice and one site in resilient mice within the BLA 2 days following CSDS with another seven differentially edited sites identified in susceptible mice and six sites in resilient mice 8 days following CSDS within this region (**Table 1**). Dynamic regulation of editing within the *Zfp324* transcript was observed within the BLA of susceptible mice with decreased and increased editing observed at 2 and 8 days, respectively. No other persistent changes were observed in this region. Within the mPFC, four sites were differentially edited in susceptible mice and another four sites were differentially edited in resilient mice 2 days following CSDS with a further seven sites in susceptible mice and four sites in resilient mice differentially edited 8 days following CSDS (**Table 2**). Increased editing at the *Commd2* locus encoding the Copper metabolism Murr1 domain-containing protein 2 (COMMD2) was observed at both 2 and 8 days following CSDS with no other persistent changes observed. Moreover, RNA editing of a nonsynonymous recoding site within the *Nova1* transcript encoding the RNA binding protein (RBP) NOVA Alternative Splicing Regulator 1 (NOVA1) was also observed in both the BLA and mPFC 8 days following CSDS, although in opposite directions. Differential editing of several sites following CSDS did not alter mRNA expression levels of candidate transcripts in either brain region (**Figure 3**).

**Table 1:** Differentially editied loci within the basolateral amygdala (BLA) following chronic social defeat stress (CSDS).

Gene name	Edit site location	Edit % CON	SEM CON	Edit % SUS	SEM SUS	Edit % difference SUS	*p* value SUS	Edit % RES	SEM RES	Edit % difference RES	*p* value RES
BLA 2 d CON vs. SUS											
*Htr2c* (site C)	chrX:143604236	22.902	0.958	25.718	0.317	2.816	0.005	24.957	0.234	2.055	0.128
*Htr2c* (site D)	chrX:143604241	58.005	0.627	60.312	0.572	2.306	0.021	59.048	0.653	1.043	0.299
*Zfp324*	*chr7:13557536*	16.870	1.008	14.172	0.635	−2.698	0.031	13.263	1.282	−3.607	0.056
*Gabra3*	chrX:69690631	89.342	0.362	90.279	0.246	0.937	0.044	89.567	0.634	0.226	0.747
BLA 2 d CON vs. RES											
*Gla*	*chrX:131123629*	3.590	0.736	5.317	0.909	1.727	0.210	6.121	0.689	2.530	0.046
BLA 8 d CON vs. SUS											
*Zfp324*	*chr7:13557536*	13.305	0.557	15.761	0.582	2.456	0.009	13.596	2.069	0.291	0.849
*Copa*	chr1:174022479	4.663	0.100	4.299	0.081	−0.364	0.016	4.556	0.074	−0.107	0.552
*Tcp11l1*	*chr2:104521242*	16.440	0.616	18.758	0.567	2.318	0.017	17.583	0.451	1.143	0.313
*Gria4*	chr9:4456006	91.448	1.531	86.767	1.210	−4.680	0.035	87.457	0.697	−3.991	0.161
*Fubp3*	*chr2:31471414*	6.945	0.257	6.087	0.273	−0.858	0.040	7.249	0.289	0.303	0.528
*Qpctl*	*chr7:19725738*	9.246	0.284	10.356	0.406	1.110	0.040	9.674	0.366	0.429	0.432
*Nova1*	chr12:47801321	12.654	0.441	11.435	0.321	−1.218	0.049	10.818	0.201	−1.835	0.038
BLA 8 d CON vs. RES											
*Bri3bp*	*chr5:125936975*	74.982	0.324	73.838	0.957	−1.144	0.253	77.648	0.162	2.665	0.001
*Slc35e1*	*chr8:75004254*	23.534	0.452	24.349	1.537	0.815	0.598	27.133	0.748	3.599	0.003
*Samd8*	*chr14:22616933*	25.989	0.501	27.677	1.143	1.688	0.180	29.333	1.179	3.344	0.012
*Nt5dc3*	*chr10:86299972*	2.464	0.085	2.340	0.089	−0.124	0.333	2.103	0.063	−0.361	0.037
*Nup155*	*chr15:8109489*	52.694	0.924	54.684	1.203	1.990	0.206	56.497	0.412	3.803	0.039

Microfluidics-based multiplex polymerase chain reaction and deep sequencing (mmPCR-seq) identified differential editing at numerous editing sites within the BLA at both 2 and 8 days following CSDS. No persistent changes in editing levels were observed at both 2 and 8 days following CSDS. Underlined edit site locations indicate RNA editing sites within exons. Italicized edit site locations indicate RNA editing sites within 3′UTRs. p < 0.05, Student’s t test.

CON, control; SUS, susceptible; RES, resilient.

**Table 2:** Differentially editied loci within the medial prefrontal cortex (mPFC) following CSDS.

Gene name	Edit site location	Edit % CON	SEM CON	Edit % SUS	SEM SUS	Edit % difference SUS	*p* value SUS	Edit % RES	SEM RES	Edit % difference RES	*p* value RES
mPFC 2 d CON vs. SUS											
*Commd2*	*chr3:57448409*	58.522	2.379	67.992	2.120	9.470	0.009	63.454	0.666	4.932	0.186
*Rsad1*	*chr11:94401990*	5.970	2.226	13.520	1.520	7.551	0.011	9.871	2.994	3.901	0.329
*Wipi2*	*chr5:143145189*	58.787	2.598	50.223	2.574	−8.564	0.034	53.321	1.579	−5.465	0.193
*Zfp81*	chr17:33472367	2.595	0.755	0.806	0.356	−1.789	0.036	1.347	1.079	−1.248	0.364
mPFC 2 d CON vs. RES											
*Ncl*	*chr1:88244312*	27.480	0.792	30.468	1.239	2.988	0.074	31.555	1.045	4.076	0.013
*Snhg11*	chr2:158209361	21.319	0.392	23.068	1.234	1.749	0.239	24.089	0.775	2.770	0.005
*Acan*	chr7:86242858	3.867	0.336	4.053	0.840	0.187	0.853	2.069	0.306	−1.798	0.007
*Nt5dc3*	*chr10:86299972*	2.380	0.070	2.367	0.230	−0.013	0.962	2.628	0.065	0.248	0.048
mPFC 8 d CON vs. SUS											
*Rn45s*	*chr17:39980697*	22.675	1.391	16.562	1.606	−6.113	0.017	20.696	0.906	−1.979	0.382
*Iqgap1*	*chr7:87856938*	4.055	0.938	8.971	1.192	4.916	0.009	4.685	2.215	0.630	0.762
*Commd2*	*chr3:57448409*	59.534	1.400	64.514	1.180	4.980	0.019	62.746	2.209	3.212	0.241
*Klf16*	*chr10:80030104*	9.441	0.346	10.741	0.329	1.300	0.020	10.037	0.560	0.595	0.372
*Rwdd2b*	*chr16:87434377*	17.327	1.261	14.255	0.554	−3.072	0.038	18.937	4.031	1.610	0.632
*Nova1*	chr12:47801321	10.549	1.160	13.241	0.374	2.691	0.038	12.090	0.446	1.541	0.401
*Dagla*	*chr19:10320223*	3.144	0.253	2.450	0.151	−0.695	0.033	2.536	0.153	−0.609	0.157
mPFC 8 d CON vs. RES											
*Rabl5*	*chr5:137388969*	16.523	0.454	15.679	0.455	−0.844	0.184	14.702	0.318	−1.821	0.045
*Copa*	chr1:174022479	4.310	0.140	4.301	0.096	−0.009	0.955	4.944	0.154	0.634	0.036
*Dnajc18*	*chr18:35834187*	12.818	0.434	13.277	0.512	0.459	0.528	14.915	0.687	2.098	0.025

Microfluidics-based multiplex PCR and deep sequencing (mmPCR-seq) identified differential editing at numerous editing sites within the PFC at both 2 and 8 days following CSDS. Increased editing at a site within the Commd2 transcript was observed at both 2 and 8 days following CSDS with no other persistent changes observed. Underlined edit site locations indicate RNA editing sites within exons. Italicized edit site locations indicate RNA editing sites within 3′UTRs. p < 0.05, Student’s t test.

CON, control; SUS, susceptible; RES, resilient.

**Figure 4:**
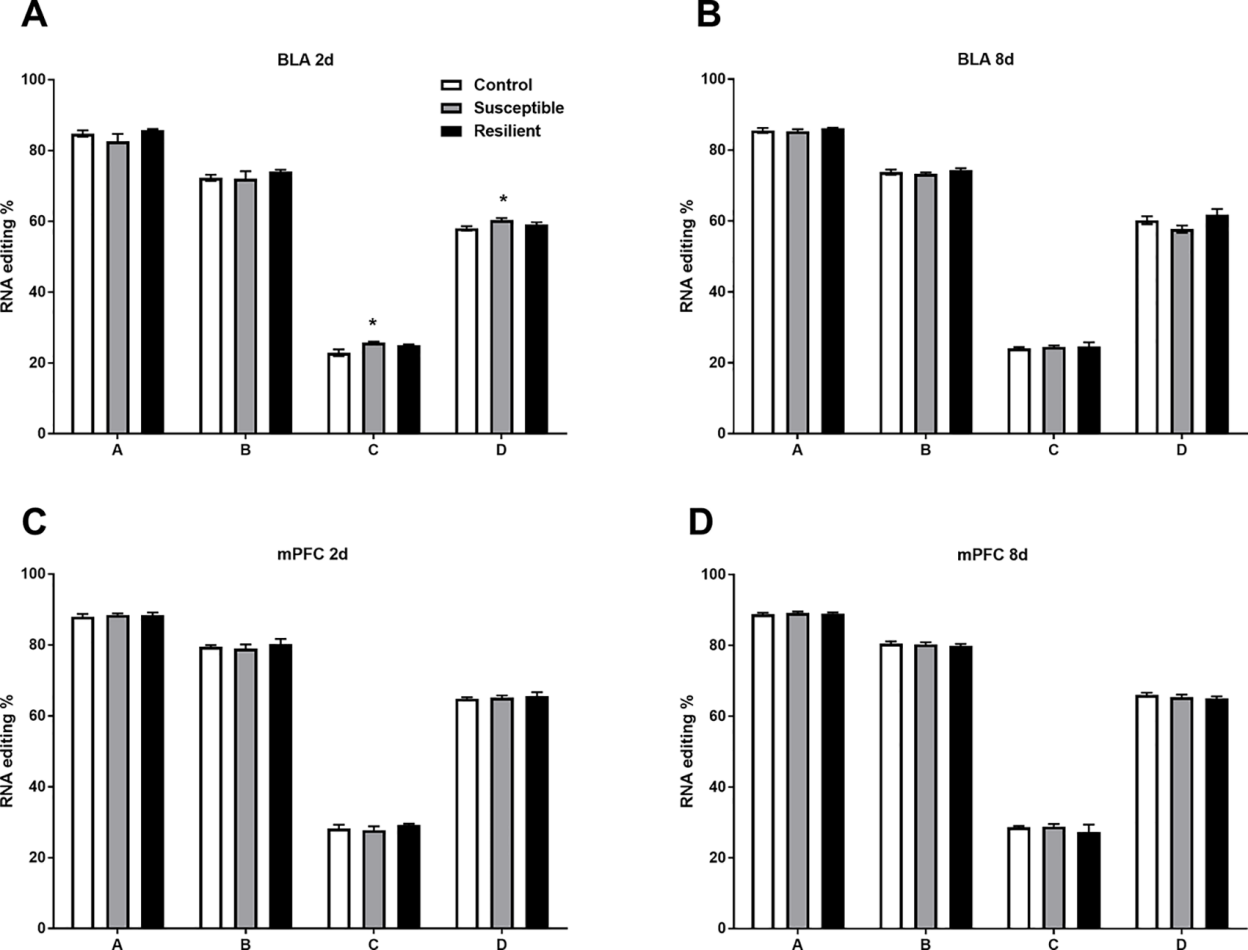
RNA editing of the *Htr2c* transcript is altered within the BLA of susceptible mice 2 days following CSDS. Sequencing analysis revealed modest increases of RNA editing at *Htr2c* sites C and D within the BLA of susceptible but not resilient mice 2 days following CSDS **(A)** with no other changes observed at 8 days **(B)** or at 2 days **(C)** or 8 days within the mPFC **(D)**. Control 2 days, *n* = 6–8; susceptible 2 days, *n* = 10; resilient 2 days, *n* = 4; control 8 days, *n* = 7–8; susceptible 8 days, *n* = 7; resilient 8 days, *n* = 3. *p < 0.05, one-way ANOVA with Sidak *post hoc* comparisons.

Well-established A-to-I editing of five sites within the *Htr2c* transcript, known as A, B, C, D, and E, results in the generation of 32 mRNA variants generating up to 24 different protein isoforms of the HTR2C receptor with varying biochemical properties (28). As expected, we detected high-confidence editing at sites A, B, C, and D but only minimal editing at site E within the mouse BLA and mPFC (**Figure 4**). CSDS induced a modest increase in editing at both the C and D sites within the BLA of susceptible mice but not resilient mice 2 days following the final defeat (**Figure 4A**) with no other changes observed at 8 days (**Figure 4B**) or within the mPFC at either time point (**Figure 4C and D**).

**Figure 5:**
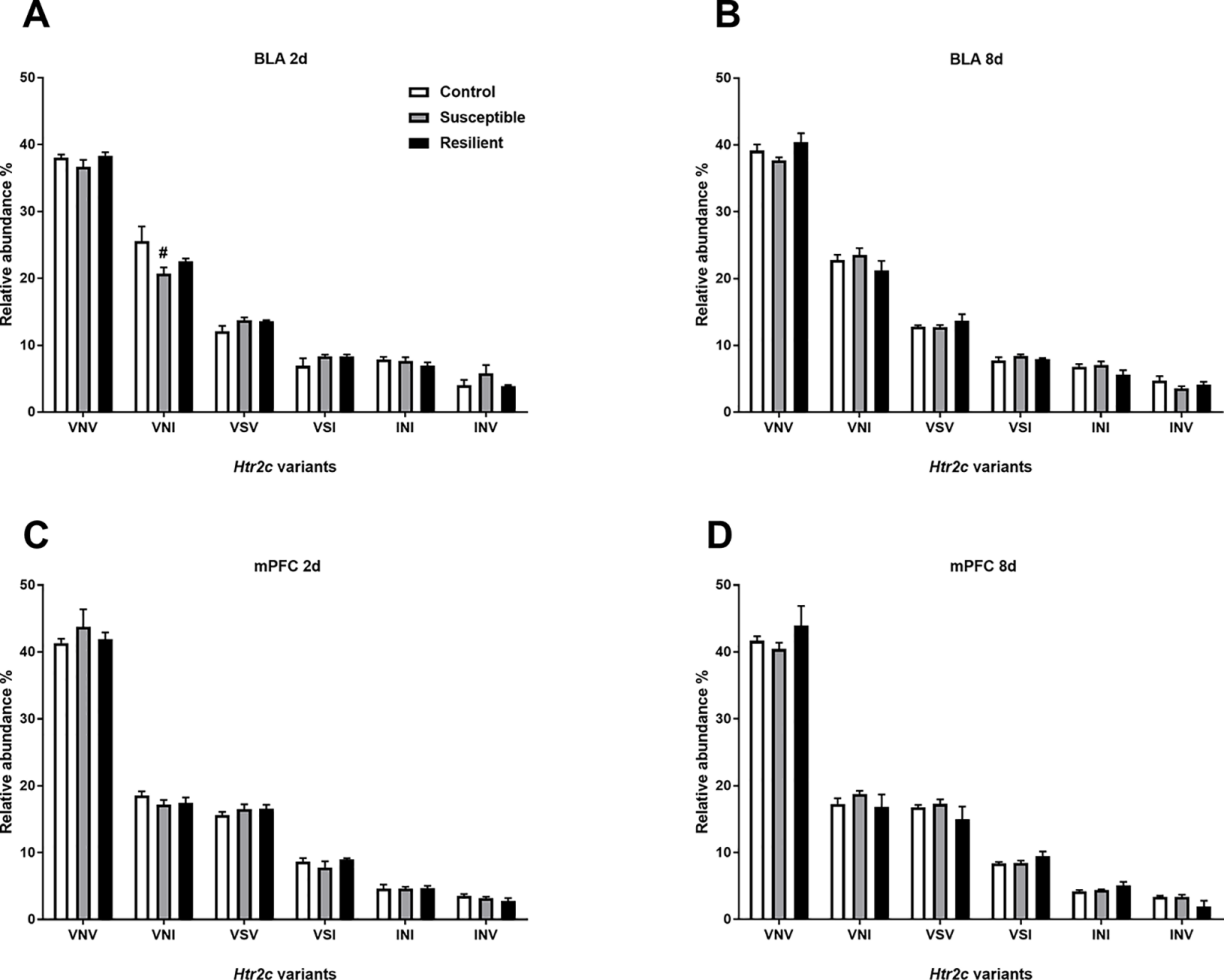
Relative abundance of *Htr2c* variants is minimally altered within the BLA 2 days following CSDS. CSDS induced a nominally significant decrease of the VNI *Htr2c* transcript variant in the BLA of susceptible mice 2 days following CSDS **(A)** with no other changes observed at 8 days **(B)** or at 2 days **(C)** or 8 days within the mPFC** (D)**. Control 2 days, *n* = 6–8; susceptible 2 days, *n* = 10; resilient 2 days, *n* = 4; control 8 days, *n* = 7–8; susceptible 8 days, *n* = 7; resilient 8 days, *n* = 3. ^#^ *p* = 0.058, one-way ANOVA with Sidak *post hoc* comparisons.

Editing at these loci induces recoding events, ultimately generating different HTR2C protein isoforms. Thus, we next quantified the relative abundance of *Htr2c* mRNA variants within the BLA and mPFC following CSDS. As expected, there was a similar distribution of *Htr2c* mRNA variants within the BLA and mPFC, with the edited VNV isoform being most abundant in both regions (**Figure 5**), as previously reported in the rodent brain (27). Following CSDS, we observed a trend toward an effect of treatment for the VNI variant [*F*_(2,18) =_ 3.068, *p* = 0.071] mainly due to a trend toward decreased VNI abundance in susceptible mice at this time point (*p* = 0.058, **Figure 5A**). No other changes were observed within the BLA at 8 days (**Figure 5B**) or within the mPFC (**Figure 5C and D**).

## Discussion

In this study, we demonstrated that CSDS in adult mice induces a moderate degree of differential editing in a subset of novel transcripts within the BLA and mPFC, including modest regulation of editing within the *Htr2c* transcript and thus isoform abundance previously demonstrated to be sensitive to stress-induced regulation. Our results further emphasize the sensitivity of RNA editing to stress and suggest that both acute and chronic changes in editing, although moderate, may contribute to behavioral deficits observed following CSDS in adult mice. Moreover, differential regulation in susceptible and resilient mice suggests that RNA editing may be a novel molecular mechanism involved in resiliency and susceptibility in this model requiring further investigation.

To our knowledge, this is the first study to investigate a large subset of RNA editing sites within the mouse brain in response to chronic stress and the first report of A-to-I editing following CSDS. Interestingly, minimal changes in transcripts encoding ADAR enzymes were identified following CSDS. Specific reduction of the interferon-inducible Adar variant 2 (p150) and Adarb1 mRNA levels was observed within the mPFC of susceptible mice only without changes in global RNA editing in these mice. RNA editing within the rodent brain is relatively stable upon induction of ADAR p150, such that decreased levels within the mPFC are unlikely to explain observed effects in this study (29). As ADARB1 is primarily responsible for editing at recoding sites (5), decreased expression in susceptible mice may mediate decreased editing at specific ADARB1 target sites. However, we observed both increased editing within *Commd2* and *Rsad1* and decreased editing in *Wipi2* and *Zfp81* within the mPFC of susceptible mice. Moreover, editing differences were also observed following CSDS in the absence of Adar mRNA expression changes, suggesting that differential RNA editing is unlikely to be mediated by the Adarb1 mRNA expression changes observed. This is in line with evidence suggesting complex regulation of RNA editing activity in a tissue-specific and cell-type-specific manner independent of ADAR family expression levels including interaction with RBPs, such as fragile X mental retardation protein (2, 5, 30, 31). Thus, our results suggest that differential RNA editing at the sites identified in this study is more likely mediated by site-specific regulation of editing activity opposed to CSDS-induced changes in the levels of ADAR family enzymes.

Although we identified differential editing at novel editing sites within the mouse brain, notable differential editing was observed within the *Htr2c* transcript specifically within the BLA of susceptible mice. Considering the well-established regulation of editing within this transcript in rodent models of acute and chronic stress as well as within the brain of MDD patients (13–16, 32), these results further implicate editing at this transcript in susceptibility to CSDS and support the model’s relevance to stress-related psychiatric disorders. However, it must be noted that stress-induced editing of the *Htr2c* transcript and variant abundance is context-dependent based on species, strain, stress modality, brain region, and developmental age (27, 28). RNA editing of the *Htr2c* transcript reduces both receptor/Gαq-protein coupling and constitutive activity of the 5HT_2C_ receptor (12, 33, 34). Transgenic mice exclusively expressing the fully edited VGV isoform also display anxiogenic and aggressive behaviors, with altered 5-HTR_2C_ receptor signaling within discrete brain regions in these mice (35). Thus, editing at this locus may mediate altered 5HTR_2C_ signaling within the BLA of susceptible mice following CSDS, yet further investigation is needed to assess the functional consequences of CSDS-induced editing at the *Htr2c* locus in this context.

Editing within transcripts encoding Gamma-Aminobutyric Acid Type A (GABA_A_) and AMPA receptor subunits were also affected following CSDS. Differential editing of sites in the *Gabra3* and *Gria4* transcripts were identified within the BLA 2 and 8 days following CSDS, respectively. The *Gabra3* transcript encodes the α3 GABA_A_ receptor subunit with editing at this highly conserved site resulting in an isoleucine-to-methionine change in the third transmembrane domain. This site is developmentally regulated and mediates receptor trafficking and GABA sensitivity whereby increased editing is thought to decrease GABA_A_ receptor signaling (36, 37). Editing at this site was also increased following chronic mild stress within the PFC of adult female rats, suggesting that this site may be sensitive to various stress modalities in different contexts (27). Moreover, modulation of GABA_A_ signaling within the BLA impairs SI in rats (38). Alterations in GABA_A_ signaling *via* RNA editing may contribute to the social avoidance phenotype observed in susceptible mice in this model, although the functional consequences of stress-induced *Gabra3* editing need to be established following CSDS.

Glutamatergic signaling may also be affected by stress-induced RNA editing as increased editing within the *Gria4* transcript encoding the AMPAR α4 subunit in the BLA of susceptible mice at 8 days is indicative of more persistent changes in the RNA editome. Editing at this *Gria4* site confers differences in AMPA receptorchannel kinetics due to regulation of *Gria4* splicing variants, which is sensitive to neuronal stimulation in rat primary cortical neurons (39). Thus, decreased editing in susceptible mice may mediate AMPAR signaling deficits in the BLA in part *via* RNA editing. Indeed, differential AMPAR signaling has been reported within the PFC (40) and hippocampus (41) following CSDS in mice. Further work is required to assess the role of AMPAR signaling, as well as GABAergic signaling, in stress susceptibility following CSDS.

Apart from changes in the aforementioned established editing sites, we aimed to identify novel editing sites sensitive to stress-induced regulation following CSDS. One such example is the *Nova1* transcript encoding the RBP NOVA1. Editing at this *Nova1* recoding site results in a serine-to-glycine exchange, which stabilizes the NOVA1 protein by decreasing proteasome-mediated degradation (42). Interestingly, we identified modest changes in *Nova1* editing in both the BLA and mPFC 8 days following CSDS with increased and decreased editing observed, respectively. Within the BLA, *Nova1* was similarly edited in both susceptible and resilient mice with differential editing only observed within the mPFC of susceptible mice, suggesting that *Nova1* editing is regulated in a region-specific manner following chronic stress. Considering the effects of RNA editing upon NOVA1 protein stability and the lack of CSDS-induced changes in *Nova1* mRNA levels, it would be of interest to assess NOVA1 protein levels within the BLA and mPFC following CSDS. Moreover, considering the established role of NOVA1 as an important RBP within the brain, which mediates both alternative splicing (43) and miRNA activity (44), NOVA1 is an interesting novel candidate requiring further investigation for its role in stress-induced regulation of the transcriptome.

Apart from such nonsynonymous recoding sites, the majority of differential editing sites identified in the current study were located within the 3′UTR of various transcripts such that the protein coding capacity of these mRNAs remains unaltered. However, RNA editing within 3′UTRs regulates mRNA availability and translation efficiency due to the editing of miRNA binding sites, which can induce differential miRNA-mediated regulation of edited transcripts (6). Persistent increases in editing at a site within the 3′UTR of *Commd2* in the mPFC of susceptible mice did not, however, alter mRNA levels in this brain region, suggesting that miRNA activity at this site is likely unaffected by editing at the site examined in this context. Further studies are required to investigate the consequences of CSDS-induced editing in 3′UTRs in this study including those sites identified in resilient mice such as in the 3′UTR of *Ncl* encoding the eukaryotic nucleolar phosphoprotein Nucelolin, which interestingly interacts with the brain-specific small nucleolar RNA MBII-52, known to regulate *Htr2c* editing within the mammalian brain (45). Furthermore, many differentially edited sites were identified within transcripts encoding proteins with poorly understood functions, particularly in resilient mice (e.g., *Bri3bp* and *Slc35e1*), which should be the focus of further investigation.

Several caveats to this study must be noted, including the small sample size for resilient groups as well as the mainly modest changes in editing levels observed. Repetition of mmPCR-seq analysis in a larger CSDS cohort would likely enable identification of further stress-sensitive editing sites, particularly those associated with resiliency. Moreover, utilizing RNA-seq would enable transcriptome-wide analysis of stress-induced editing and mRNA levels. Despite this, the A-to-I editing changes observed in this study are similar with the editing level changes observed in the rat PFC and amygdala following chronic stress employing a similar mmPCR-seq technique (27). Such modest changes of RNA editing in bulk brain tissue are also likely explained by cellular heterogeneity as recent advances in single-cell transcriptomics have demonstrated that A-to-I editing is indeed cell type specific with changes even observed between different cells of given cellular population within the mammalian brain (46, 47). Thus, it would be of interest in the future to study stress-induced changes in RNA editing using single cell transcriptomics.

In conclusion, the current study has identified A-to-I editing as another molecular mechanism of likely relevance to stress resiliency and susceptibility to CSDS in adult mice, in line with the growing appreciation for stress-induced regulation of RNA metabolism within the brain (21, 48, 49). Further investigation of the consequences of these editing changes is required at both the mRNA and protein levels to decipher the functional consequences of RNA editing following chronic stress.

## Ethics Statement

This study was carried out in accordance with the recommendations of the European Communities; Council Directive 2010/63/EU. The protocols were approved by the Animal Care and Use Committee of the Government of Upper Bavaria, Munich, Germany.

## Author Contributions

AD, KK, EL, and AC conceptualized and designed the experiments and wrote the manuscript. AD and EP conducted all animal experiments analyzed by AD. AD and KK conducted and analyzed all mmPCR-seq experiments. AD and FS conducted qPCR experiments analyzed by AD.

## Conflict of Interest Statement

The authors declare that the research was conducted in the absence of any commercial or financial relationships that could be construed as a potential conflict of interest.
